# Cognitive function trajectories and their determinants in older people: 8 years of follow-up in the English Longitudinal Study of Ageing

**DOI:** 10.1136/jech-2017-210116

**Published:** 2018-04-24

**Authors:** Paola Zaninotto, G David Batty, Michael Allerhand, Ian J Deary

**Affiliations:** 1 Research Department of Epidemiology and Public Health, University College London, London, UK; 2 Centre for Cognitive Ageing and Cognitive Epidemiology, Department of Psychology, University of Edinburgh, Edinburgh, UK

**Keywords:** ageing, longitudinal studies, cognition

## Abstract

**Background:**

Maintaining cognitive function is an important aspect of healthy ageing. In this study, we examined age trajectories of cognitive decline in a large nationally representative sample of older people in England. We explored the factors that influence such decline and whether these differed by gender.

**Methods:**

Latent growth curve modelling was used to explore age-specific changes, and influences on them, in an 8-year period in memory, executive function, processing speed and global cognitive function among 10 626 participants in the English Longitudinal Study of Ageing. We run gender-specific models with the following exposures: age, education, wealth, childhood socioeconomic status, cardiovascular disease, diabetes, physical function, body mass index, physical activity, alcohol, smoking, depression and dementia.

**Results:**

After adjustment, women had significantly less decline than men in memory (0.011, SE 0.006), executive function (0.012, SE 0.006) and global cognitive function (0.016, SE 0.004). Increasing age and dementia predicted faster rates of decline in all cognitive function domains. Depression and alcohol consumption predicted decline in some cognitive function domains in men only. Poor physical function, physical inactivity and smoking were associated with faster rates of decline in specific cognitive domains in both men and women. For example, relative to study members who were physically active, the sedentary experienced greater declines in memory (women −0.018, SE 0.009) and global cognitive function (men −0.015, SE 0.007 and women −0.016, SE 0.007).

**Conclusions:**

The potential determinants of cognitive decline identified in this study, in particular modifiable risk factors, should be tested in the context of randomised controlled trials.

## Introduction

Decline in cognitive function is a major concern for older adults.^1^ Lower cognitive function and cognitive decline are also associated with an increased risk of mortality, disability and poor quality of life.^2 3^ While it is well documented that cognitive functioning in general declines in older age,^4–6^ there is a suggestion that different domains of cognition decline at different rates. Crystallised intelligence, for instance, as denoted by verbal ability, general knowledge and number skills, is more likely to endure with age.^6^ Other cognitive abilities, such as memory, executive function and processing speed, known collectively as fluid intelligence, however, show, on average, a greater degree of decline^7^ which may occur from as early as middle age.^5^ Understanding the age-related cognitive decline and the factors that potentially mitigate such decline is important for early interventions.^3^

A growing body of evidence is emerging with regard to predictors of cognitive decline in older age. The most commonly investigated being sociodemographic, health, depression and health behaviour factors.^3 8–10^ Depression has been consistently associated with faster cognitive decline.^10 11^ Poor health status, assessed in a variety of ways, has been related to cognitive decline.^3 9 12 13^ Among the health-related behaviours, physical inactivity and current or ever smoking status have been associated with steeper cognitive decline.^3 14 15^

Research on the relationship between sociodemographic factors, such as sex, socioeconomic status, education and cognitive function decline, has yielded mixed evidence. With respect to sex differences in rates of change in cognitive abilities, empirical evidence has not been conclusive, with some reporting no differences^16^ and others showing steeper decline in men than women for specific cognitive domains.^17^ Recent studies on education and cognitive decline yielded to the consistent finding that education contributes to the initial levels of cognitive function but does not influence age-related cognitive decline.^9 18^ Studies on the role of childhood and/or current socioeconomic status on cognitive decline are mixed, with some reporting a faster decline among disadvantaged individuals^19^ and others reporting no associations.^9^

Longitudinal studies which collect a broad range of factors and characterise change in cognitive functions over time in older age are best placed to provide insights into age-related decline and the factors contributing to such decline, which both remain the subject of debate.^8 20 21^ In recent years, prospective studies of ageing in multiple countries have emerged, designed to be comparable with the Health and Retirement Study (HRS)^22^ in the USA, including the English Longitudinal Study of Ageing (ELSA),^23^ which we describe in the present manuscript. All of these large nationally representative studies have included measures of cognitive abilities that assess brain functioning across several domains, such as memory, executive function and processing speed. The studies were also designed to cover social, economic, behavioural and health aspects of ageing and, therefore, are well suited to explore potential predictors of cognitive decline. However, to date, none of the above-mentioned national ageing studies have explored a broad range of predictors of cognitive decline. ELSA was the first study initiated to be comparable with HRS, therefore offering the longest follow-up among these ageing studies.

Accordingly, the aim of this study was to examine age trajectories of cognitive function and influences on them in a large nationally representative sample of older people living in England. Trajectories of cognitive function are evaluated with the use of three important domains (memory, executive function and processing speed), characterising fluid intelligence over an 8-year period. Potential predictors of cognitive decline were explored separately for men and women and carefully selected from the literature to cover several broad categories: demographic (age), socioeconomic status (education, wealth and childhood socioeconomic status), health (cardiovascular disease (CVD) and diabetes), physical functioning (limitations with activities of daily living (ADL) and walking difficulties), health behaviours (body mass index (BMI), physical activity, alcohol and smoking), depression and dementia.

## Methods

### Data sources

Data are drawn from ELSA which has been described in detail elsewhere.^23^ In brief, a representative sample of 11 391 people aged 50 years and over living in private households in England who had previously participated in the Health Survey for England (wave 0, 1998, 1999 or 2001) was interviewed every 2 years. We used data up to wave 5 (2010–2011). ELSA was conducted in accordance with the Declaration of Helsinki, and ethical approval and experimental protocols were granted by the Multicentre Research and Ethics Committee. Participants gave their informed consent to take part in the study.

### Measures

Cognitive function was assessed at each wave using a battery of standard tests covering three major cognitive domains: memory, processing speed and executive function. *Memory* was measured using a word-list learning test in which a list of 10 words was presented orally to study participants who were then asked to recall as many words as possible immediately after the reading of list had been completed and then again after around a 5 min delay during which they completed other survey questions. The word list comprises four different versions, so that different lists can be administered at different waves of data collection. We computed an overall memory score (ranging from 0 to 20) by adding the points of the immediate and delayed recall tests (maximum of 10 points for immediate and 10 points for delayed recall; correlation coefficient of 0.70). *Executive function* was measured using a test of how quickly participants could name as many different animals as possible in 1 min (semantic verbal fluency). The overall score in the sample ranged from 0 to 60. *Processing speed* was measured using a letter cancellation test. The participant was handed a clipboard to which was attached a page of random letters of the alphabet set out in rows and columns and was asked to cross out as many target letters (P and W) as possible within 1 min. The total number of letters searched (score 0 to 64) provided a measure of speed of processing.

All scores were normally distributed; there was no evidence of floor and ceiling effect.

Age, sex, wealth, education and childhood socioeconomic positions were measured at baseline (2002–2003). Total wealth was defined as the sum of financial, physical (eg, business and land) and housing wealth, minus debts, from which we computed tertiles (high, medium and low). Education was categorised into high (college/university and above), medium (advanced level) and low (ordinary level or lower). Three levels of childhood socioeconomic status were derived from paternal occupation at the age of 14 years (high (managerial, professional and administrative occupations or business owners), intermediate (trade-related and services-related occupations) and low (manual and casual occupations and other occupations)).

CVD, diabetes and dementia were assessed at each wave using self-reported doctor diagnosis. Participants were classified as having one or more limitations with ADL if they reported having difficulties in performing any of the six activities (eg, dressing, walking across a room, bathing or showering, eating, getting in/out of bed and using the toilet). Poor mobility was assessed by asking respondents if they had any difficulties walking 100 yards (91.44 m). Depression was measured using the 8-item version of the Centre for Epidemiologic Studies—Depression scale^24^ with a cut-off of four or more depressive symptoms.^25^ Self-reported health behaviours included smoking status (non-smoker and current smoker), frequency of alcohol consumption in the past year (less than daily and daily) and physical activity during leisure time, recorded as participation in vigorous, moderate and mild activities (active and sedentary). BMI was derived from height and weight measured by a nurse at waves 0, 2 or 4. Gait speed was assessed by two-timed walks at normal pace, each of 8 feet, among participants aged 60 years and over.

### Statistical analyses

The analytical sample comprised participants who had answered the cognitive function tests, consisting of 10 626 (5777 females) at wave 1 (93% of 11 391 study participants), 8348 at wave 2 (95% of 8780), 6951 at wave 3 (92% of 7535), 5685 at wave 4 (86% of 6623) and 5512 at wave 5 (88% of 6242). To examine trajectories of change over time in cognitive function, we used linear latent growth curve (LGC) methodology in Mplus V.7^26^ with Full Information Maximum Likelihood (FIML) algorithm for unbalanced data.^27^ FIML has the benefit of computing parameter estimates on the basis of all available data–without either imputing or dropping data when missing–under the assumption that data are missing at random. However, attrition due to mortality or dropout can violate this assumption. Therefore, we further explored how sensitive the model’s parameter estimates were to missing values using imputed data^28^ and found that the sign and significance of model parameters was the same across multiple datasets; therefore, we judged the occurrence of missing data to be ignorable.

To facilitate comparisons, we standardised the cognitive function scores to have a mean equal to 0 and an SD equal to 1. We fitted one model for each of the cognitive function domains. Sociodemographic characteristics were modelled as time invariant factors. For CVD, diabetes, ADL, walking difficulties, depression, smoking, alcohol and physical activity, we used information from all five waves to indicate whether the responded ever reported the condition (no and yes). For BMI, we computed the average reported at waves 0, 2 or 4. Gait speed was computed as the average reported during the study period.

Risk factors were entered simultaneously in each model of the cognitive function domains. In the models with risk factors, we found that women had significantly slower rate of change in memory, executive and global cognitive function than men (ST1). Furthermore, we tested for gender/covariate interactions and found some evidence of gender differences in intercepts and slopes. Results of risk factors are reported by gender.

In our sample, there was minimal evidence of practice effects between the first and second occasion and no evidence of practice effects between the second and subsequent occasions. Therefore, retest was not included in the statistical models.^29^

Changes in global cognitive function were estimated using a latent construct; thus, on each occasion, memory, executive function and processing speed were modelled as indicators of global cognitive function. Therefore, we assessed factorial invariance by evaluating whether the same construct (eg, global cognitive function) was assessed on the same metric across each measurement occasion using a hierarchy of tests (configural invariance, metric invariance, strong invariance and strict invariance) and comparing model fit.^30^ Results suggested that strong factorial invariance held across occasions; therefore, the same latent construct was identified longitudinally (results available on request).

We present ageing-vector graphs of predicted cognitive function scores in order to show visually the level of each score at baseline, direction and amount of change throughout the age range of our sample. The graphs reveal both any trends by age of the sample at baseline and cohort-specific within-person changes over time in cognitive function^31^; they were fitted using STATA V.14.

Lastly, we conducted several sensitivity analyses to further assess whether a different set of predictors of cognitive decline were found for people aged 60 years and over; and we also assessed the impact of sample attrition.

## Results

In table 1, we show the summary statistics for the predictors of cognitive decline and baseline levels of each cognitive function measured by gender. One in 3 men and 1 in 4 women reported ever being diagnosed with CVD during the study follow-up period and just over 1 in 10 with diabetes. On average, respondents in our sample were overweight, as indicated by the average BMI of 28 kg/m^2^. Over 40% of men and 27% of women reported drinking alcohol daily, whereas women were more physically inactive than men. Over one-third of women and 24% of men reported ever being depressed. About 3% of respondents were diagnosed with dementia. At baseline, respondents recalled (sum of immediate and 5 min delayed) on average 9.2 (SD 3.5) words (9.7, SD 3.6 in women), named 19.8 (SD 6.4) animals (18.9, SD 6.1 in women) and correctly identified 17.7 (SD 5.7) target letters (19.3, SD 6.1 in women).

**Table 1:** Summary of characteristics of participants: the English Longitudinal Study of Ageing, 2002–2003 to 2010–2011

	Men (n=4849)		Women (n=5777)	
Demographic				
Age, year, mean, (SD) range	64.6 (9.8)	50–100	65.0 (10.2)	50–100
Socioeconomic				
Wealth				
High	30.8	(29.5 to 32.1)	34.2***	(33.0 to 35.5)
Middle	33.6	(32.3 to 34.9)	33.9	(32.2 to 35.2)
Low	35.6	(34.2 to 36.9)	31.9***	(30.7 to 33.1)
Childhood SES				
High	27.4	(26.1 to 28.7)	29.2	(28.1 to 30.4)
Middle	32.9	(31.7 to 34.3)	31.7	(30.5 to 32.9)
Low	39.6	(38.2 to 41.0)	39.1	(37.8 to 40.3)
Education				
High	16.4	(15.4 to 17.5)	12.1***	(11.3 to 12.9)
Middle	29.5	(28.2 to 30.8)	35.4***	(34.2 to 36.7)
Low	54.1	(52.6 to 55.5)	52.5***	(51.2 to 53.8.)
Health				
CVD % (95% CI)	28.9	(27.6 to 30.2)	20.3***	(19.3 to 21.4)
Diabetes % (95% CI)	14.7	(13.8 to 15.8)	10.5***	(9.8 to 11.3)
Physical functioning				
ADL† % (95% CI)	70.0	(68.7 to 71.3)	69.5	(68.3 to 70.6)
Poor mobility‡	17.2	(16.2 to 18.3)	20.0***	(19.0 to 21.1)
Health behaviours				
BMI	27.8	(27.7 to 27.9)	27.9	(27.7 to 28.0)
Current smoker	19.2	(18.1 to 20.3)	19.3	(18.3 to 20.4)
Daily alcohol consumption	41.7	(40.3 to 43.1)	26.9***	(25.8 to 28.1)
Physically inactive	31.1	(29.8 to 32.4)	41.2***	(40.0 to 42.5)
Depression % (95% CI)	23.8	(22.6 to25.0)	36.3***	(35.1 to 37.6)
Ever dementia§	3.2	(2.7 to 3.7)	3.1	(2.7 to 3.6)

Cognitive function measures	Mean (SD)	Range	Mean (SD)	Range
Memory				
Original score	9.2 (3.5)	0–20	9.7 (3.6)***	0–20
z-Transformed score	−0.07 (0.99)	−2.7–3.0	0.06 (1.01)	−2.7–3.0
Executive function				
Original score	19.8 (6.4)	0–48	18.9 (6.1)***	0–50
z-Transformed score	0.07 (1.02)	−3.1–4.6	−0.06 (0.98)	−3.1–4.9
Processing speed				
Original score	17.7 (5.7)	0–62	19.3 (6.1)***	0–63
z-Transformed score	−0.1.4 (1.0)	−3.1–7.3	−0.12 (1.0)	−3.1–7.5

*P<0.05 for the gender difference.

**P<0.01.

***P<0.001.

†One or more limitations with ADL.

‡Difficulties walking 100 yards.

§Reported between baseline and wave 5.

ADL, activities of daily living; BMI, body mass index; CVD, cardiovascular disease; SES, socioeconomic status.

### Age trajectories of cognitive function

In table 2, we show estimated parameters of the LGC, with age (centred to the mean of 65) for each of the cognitive domains and the global cognitive function score. The fit indices suggest that the proposed models fit the data well. The intercept values refer to the mean standardised scores of each cognitive function domain of men who were aged 65 years at baseline, and the slope values refer to the average SD change for each additional wave of the study (2-year interval). For example, the average standardised baseline score of memory was −0.025 (SE 0.012) for men aged 65 years, this decreased at an average rate of −0.037 (SE 0.004) for each additional wave of the study. Initial levels of each cognitive function domain were lower for older individuals; for each year increase in age, there were a −0.038 decrease in memory score, a −0.030 decrease in executive function score, a −0.028 decrease in processing speed score and a −0.037 decrease in global cognition score. Women had higher memory (0.158, SE 0.016), processing speed (0.288, SE 0.017) and global cognitive function levels (0.099, SE 0.004) at baseline than men but lower executive function (−0.120, SE 0.017). Table 2 also shows the effect of baseline age on the rate of change of each cognitive function score. A person who is older at baseline has a more negative slope (memory −0.004, p<0.001; executive function −0.005, p<0.001; processing speed −0.003, p<0.001 and global cognitive function score −0.005, p<0.001), that is, a steeper decline per each additional wave of the study (2 years). There was no gender difference in the rate of change of memory, executive function and processing speed, whereas the positive coefficient for the global cognitive function score indicated that women had slower decline compared with men (0.013, SE 0.002, p<0.010).

**Table 2:** Results of the linear growth curve model with age and sex for each cognitive function domain

Growth parameters	Memory (standardised score)*		Executive (standardised score)*		Processing speed (standardised score)*		Global cognitive function (standardised score)*	
	Estimate (SE)	P values	Estimate (SE)	P values	Estimate (SE)	P values	Estimate (SE)	P values
Intercept	−0.025 (0.012)	<0.050	0.099 (0.013)	<0.001	−0.171 (0.013)	<0.001	0.000 (0.000)	na
Intercept variance	0.429 (0.011)	<0.001	0.512 (0.012)	<0.001	0.487 (0.012)	<0.001	0.339 (0.010)	<0.001
Slope	−0.037 (0.004)	<0.001	−0.037 (0.004)	<0.001	−0.057 (0.004)	<0.001	−0.057 (0.003)	<0.001
Slope variance	0.007 (0.001)	<0.001	0.015 (0.001)	<0.001	0.010 (0.001)	<0.001	0.006 (0.001)	<0.001
Intercept on								
Female	0.158 (0.016)	<0.001	−0.120 (0.017)	<0.001	0.288 (0.017)	<0.001	0.099 (0.004)	<0.010
Age	−0.038 (0.001)	<0.001	−0.030 (0.001)	<0.001	−0.028 (0.001)	<0.001	−0.037 (0.001)	<0.001
Slope on								
Female	0.008 (0.005)	0.158	0.009 (0.006)	0.123	0.008 (0.005)	0.121	0.013 (0.002)	<0.010
Age	−0.004 (0.000)	<0.001	−0.005 (0.000)	<0.001	−0.003 (0.000)	<0.001	−0.005 (0.000)	<0.001
Intercept and slope correlation	0.011 (0.003)	<0.001	0.002 (0.003)	0.488	−0.019 (0.003)	<0.001	0.000 (0.002)	0.967
Model fit								
CFI	0.991		0.996		0.997		0.976	
TLI	0.989		0.994		0.996		0.969	
RMSEA	0.031		0.021		0.017		0.036	
n	10 626		10 626		10 626		10 626	

*To the z-score, mean 0 and SD 1.

CFI, Comparative Fit Index; RMSEA, root mean-square error of approximation; TLI, Tucker–Lewis Index.

**Figure 1:**
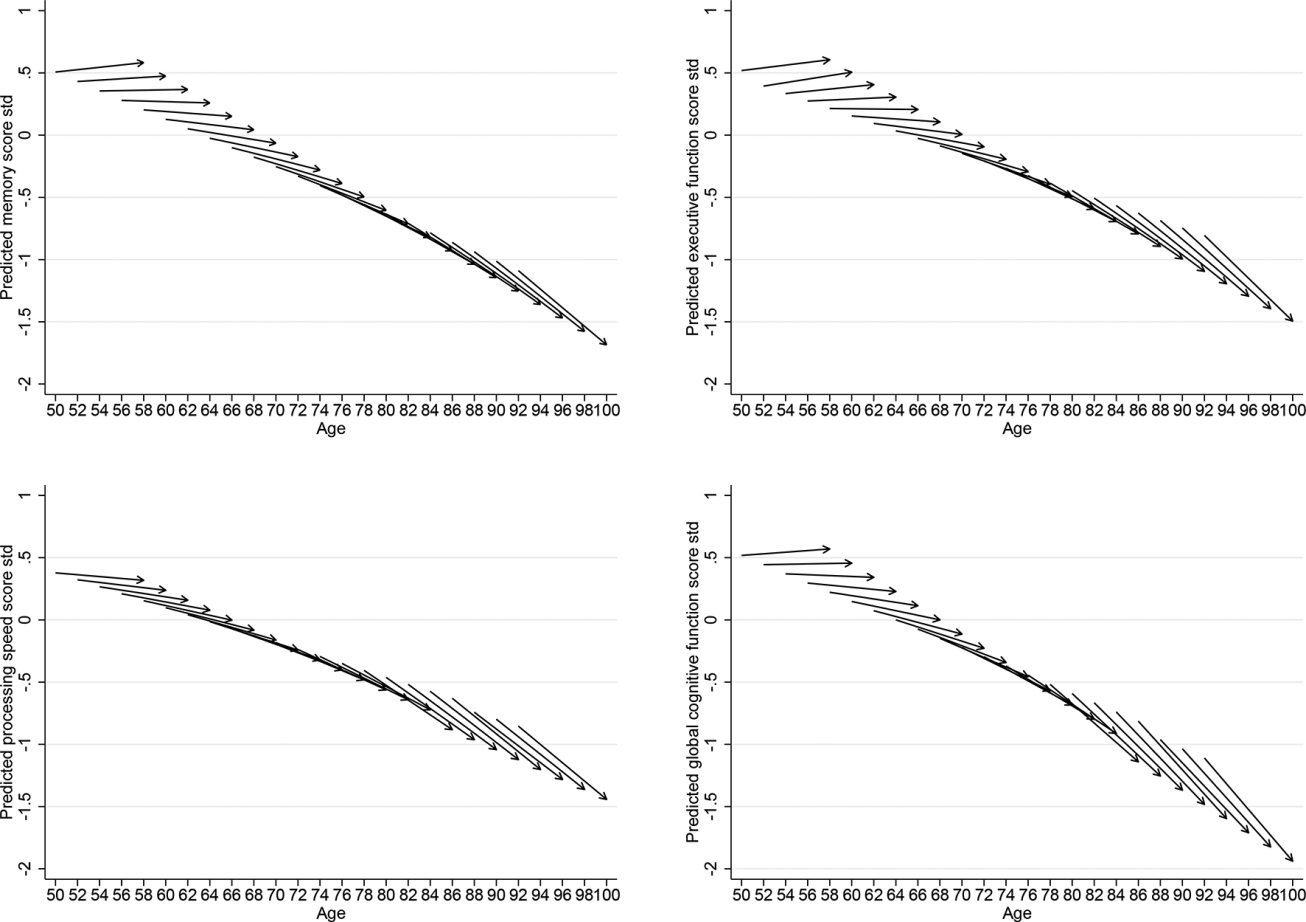
Vector graphs showing the predicted 8-year trajectories of men of memory, executive function, processing speed and global cognitive function for 2-year cohorts, English Longitudinal Study of Ageing 2002–2003 to 2010–2011.

**Figure 2:**
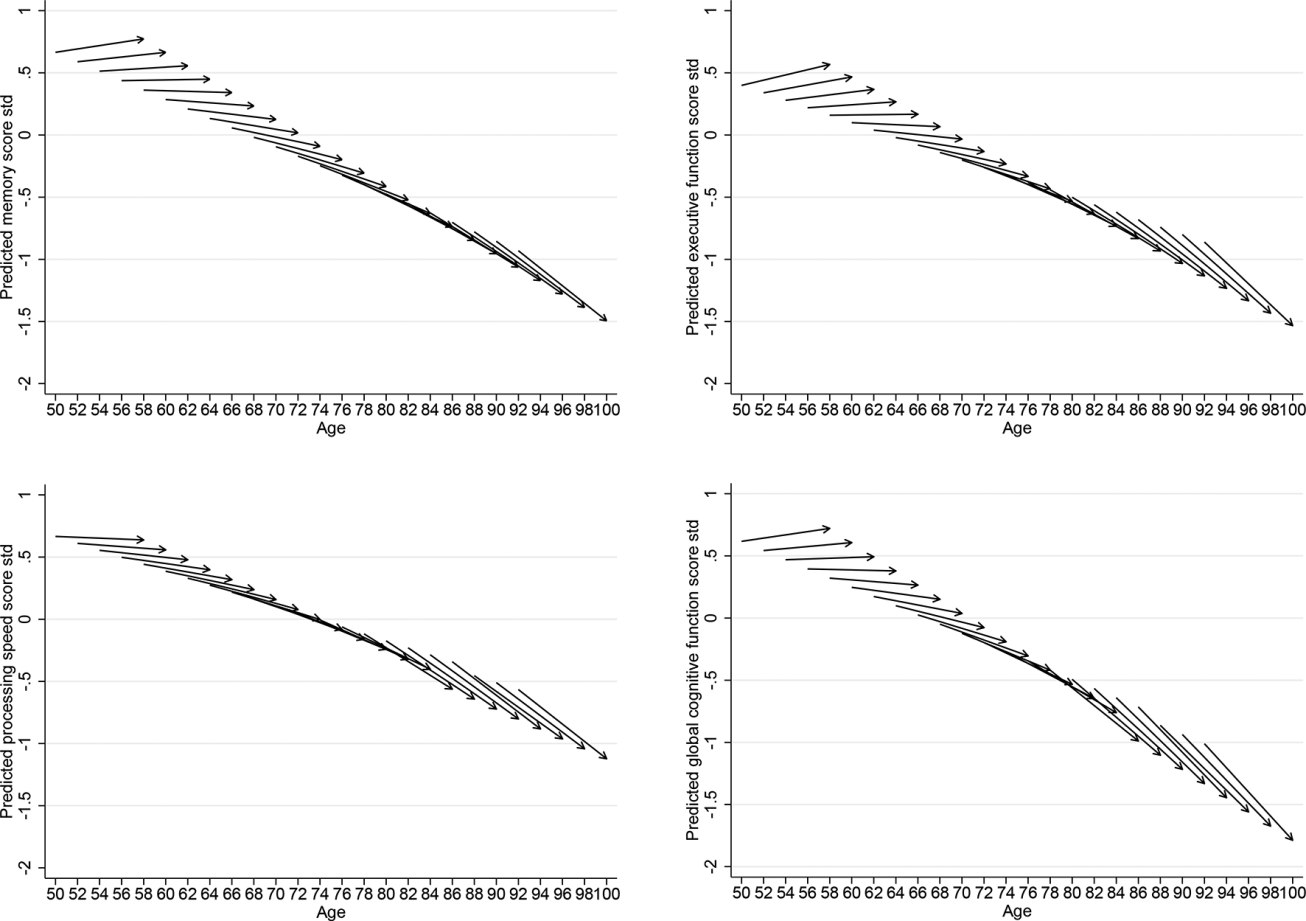
Vector graphs showing the predicted 8-year trajectories of women of memory, executive function, processing speed and global cognitive function for 2-year cohorts, English Longitudinal Study of Ageing 2002–2003 to 2010–2011.

The ageing-vector graphs shown in figures 1 and 2 provide a summary of the LGC models presented in table 2. Each arrow represents the predicted cognitive function score at baseline and change for every 2-year cohort. The horizontal axis indicates the respondent’s age at the beginning and the end of the survey period from 2002 to 2003 to 2010–11. The vertical axis indicates the respondent’s predicted scores. The figures show that for adults at older ages, there is a steeper decline over the 8-year period in each of the cognitive function domain.

### Predictors of cognitive function decline

In table 3, we report the associations between covariates and the baseline level (intercept) and change overtime (slope) in each domain of cognitive function for men. Increasing age, middle and low wealth, low childhood socioeconomic status, educational attainment, limitations with ADL, physical inactivity and dementia were related to lower baseline levels of memory, executive function, processing speed (with the exception of low childhood socioeconomic status) and global cognitive function scores. In addition, CVD was related to lower baseline memory and diabetes to lower processing speed. Higher BMI was associated with slightly higher baseline levels of executive function (0.011, SE 0.003) and global cognitive function (0.006, SE 0.002). Daily frequency of alcohol consumption was related with higher levels of baseline memory (0.081, SE 0.022), executive function (0.101, SE 0.026), processing speed (0.069, SE 0.024) and global cognitive function (0.094, SE 0.019); smoking status was associated with lower baseline levels of executive function (−0.065, SE 0.033) and processing speed (−0.072, SE 0.31). Depression was related to lower baseline executive function (−0.076, SE 0.030) and global cognitive function (−0.054, SE 0.023).

**Table 3:** Predictors of intercepts and slopes of change of each cognitive function domain in 4849 men, the English Longitudinal Study of Ageing, 2002–2003 to 2010–2011

Covariate	Memory intercept	Memory slope	Executive function intercept	Executive function slope	Processing speed intercept	Processing speed slope	Global cognitive function intercept	Global cognitive function slope
Demographic								
Age†	−0.033 (0.001)***	−0.003 (0.000)***	−0.026 (0.001)***	−0.004 (0.000)***	−0.027 (0.001)***	−0.002 (0.000)***	−0.033 (0.001)***	−0.004 (0.000)***
Socioeconomic								
Wealth (middle)†	−0.101 (0.027)***	–	−0.086 (0.031)***	–	−0.145 (0.029)***	–	−0.118 (0.023)***	–
Wealth (low)†	−0.214 (0.031)***	–	−0.198 (0.035)***	–	−0.223 (0.032)***	–	−0.236 (0.026)***	–
Childhood SES (middle)†	–	–	–	–	–	−0.020 (0.010)*	–	–
Childhood SES (low)†	−0.068 (0.029)*	–	−0.116 (0.033)***	–	–	–	−0.076 (0.025)***	
Education (middle)†	−0.145 (0.033)***	–	−0.149 (0.038)***	–	−0.131 (0.035)***	–	−0.157 (0.029)***	–
Education (low)†	−0.361 (0.024)***	–	−0.302 (0.039)***	–	−0.243 (0.036)***	–	−0.355 (0.029)***	0.020 (0.009)*
Health								
CVD‡	−0.064 (0.027)*	–	–	–	–	–	–	–
Diabetes‡	–	–	–	–	−0.079 (0.035)*	–	–	–
Physical functioning								
ADL§	−0.198 (0.025)***	–	−0.184 (0.029)***	–	−0.079 (0.026)***	–	−0.187 (0.022)***	−0.013 (0.007)*
Poor mobility‡^‡^	–	–		–	–	–	–	–
Health behaviours								
BMI¶			0.011 (0.003)**	–	–		0.006 (0.002)*	–
Current smoker‡	–	–	−0.065 (0.033)*	–	−0.072 (0.031)*	–	–	–
Daily alcohol drinking‡	0.081 (0.022)***	–	0.101 (0.026)***	–	0.069 (0.024)**	−0.017 (0.008)*	0.094 (0.019)***	–
Physically inactive‡	−0.141 (0.027)***	–	−0.116 (0.031)***	–	−0.104 (0.029)***	–	−0.139 (0.015)***	−0.015 (0.007)*
Depression‡	–	–	−0.076 (0.030)*	−0.022 (0.011)*	–		−0.054 (0.023)*	−0.020 (0.007)*
Dementia‡	−0.413 (0.064)***	−0.158 (0.027)***	−0.198 (0.073)***	−0.223 (0.031)***	−0.307 (0.069)***	−0.094 (0.028)***	−0.358 (0.055)***	−0.191 (0.021)***

***P<0.001, **P<0.01, *P<0.05.

Cognitive function domain scores are standardised to the z-score, mean 0 and SD 1. All predictors were entered simultaneously.

Cells with dashes represent non-significant effects.

†Measured at baseline.

‡Ever reported between waves 1 and 5.

§Ever reported 1+ limitations with ADL.

¶Average between wave 0, wave 2 and wave 4.

ADL, activities of daily living; BMI, body mass index; CVD, cardiovascular disease; SES, socioeconomic status.

Predictors of cognitive decline were different according to each domain (table 3). Increasing age was associated with a steeper decline in all domains of cognitive function. People in the middle childhood socioeconomic status group compared with those in the highest had significantly steeper decline in processing speed (−0.020, SE 0.010). Low educational attainment was related to a slower decline in global cognitive function (0.020, SE 0.009). Being physically inactive was related to faster decline in global cognitive function (−0.015, SE 0.007) and alcohol consumption to faster decline in processing speed (−0.017, SE 0.008). Being diagnosed with dementia was significantly related to steeper rates of longitudinal decline in memory (−0.413, SE 0.064), executive function (−0.198, SE 0.073), processing speed (−0.307, SE 0.069) and global cognitive function (−0.358, SE 0.055). Those who reported being depressed had a steeper rate of decline in executive function (−0.022, SE 0.011) and global cognitive function (−0.020, SE 0.007).

In table 4, we report results for women. Childhood middle socioeconomic status was related to lower baseline memory, executive function and global cognitive function. Childhood low socioeconomic status was related to lower processing speed. CVD, diabetes and smoking were not associated with any cognitive function intercepts. Poor mobility was significantly related to lower baseline processing speed (−0.086, SE 0.031). Depression was associated with lower baseline memory (−0.049, SE 0.022), processing speed (−0.070, SE 0.025) and global cognitive function (−0.0746, SE 0.01), but not executive function.

**Table 4:** Predictors of intercepts and slopes of change of each cognitive function domain in 5777 women, the English Longitudinal Study of Ageing, 2002–2003 to 2010–2011

Covariate	Memory intercept	Memory slope	Executive function intercept	Executive function slope	Processing speed intercept	Processing speed slope	Global cognitive function intercept	Global cognitive function slope
Demographic								
Age†	−0.034 (0.001)***	−0.004 (0.000)***	−0.025 (0.001)***	−0.004 (0.000)***	−0.024 (0.001)***	−0.003 (0.000)***	−0.032 (0.001)***	−0.005 (0.000)***
Socioeconomic								
Wealth (middle)†	−0.093 (0.026)***	–	−0.078 (0.027)**	–	–	–	−0.088 (0.022)***	–
Wealth (low)†	−0.233 (0.028)***	–	−0.209 (0.030)***	–	−0.124 (0.032)***	–	−0.230 (0.024)***	–
Childhood SES (middle)†	−0.086 (0.027)**	–	−0.080 (0.028)**	–	–	–	−0.079 (0.023)**	–
Childhood SES (low)†	−0.145 (0.027)***	0.021 (0.007)*	−0.136 (0.028)***	–	−0.111 (0.031)***	–	−0.149 (0.023)***	0.020 (0.007)*
Education (middle)†	−0.112 (0.034)**	–	−0.240 (0.035)***	–	−0.088 (0.039)*	–	−0.169 (0.029)***	–
Education (low)†	−0.316 (0.035)***	−0.026 (0.012)*	−0.456 (0.036)***	–	−0.130 (0.040)**	-–	−0.369 (0.030)***	–
Health								
CVD‡	–	–	–	–	–	–	–	–
Diabetes‡	–	–	–	–	–	–	–	–
Physical functioning								
ADL§	−0.200 (0.024)***	–	−0.167 (0.025)***	–	−0.122 (0.027)***	–	−0.196 (0.020)***	−0.013 (0.006)*
Poor mobility‡	–	−0.020 (0.010)*	–	–	−0.086 (0.031)**	–	–	–
Health behaviours								
BMI¶	–	–	0.006 (0.002)**	–	–			
Current smoker‡	–	–	–	–	–	−0.025 (0.010)*	–	−0.020 (0.007)**
Daily alcohol drinking‡	0.100 (0.024)	–	0.104 (0.025) ***	–	–	–	−0.100 (0.020)***	–
Physically inactive‡	− 0.098 (0.024)***	− 0.018 (0.009) *	− 0.118 (0.025)***	–	−0.136 (0.027)***	–	− 0.123 (0.020) ***	− 0.016 (0.007) *
Depression‡	−0.049 (0.022)**	–	–	–	−0.070 (0.025)**	–	−0.046 (0.019)**	–
Dementia‡	−0.524 (0.061)***	−0.184 (0.026)***	−0.275 (0.063)***	−0.210 (0.027)***	−0.362 (0.069)***	−0.069 (0.028)*	−0.452 (0.052)***	−0.197 (0.020)***

***P<0.001, **P<0.01, *P<0.05.

†Measured at baseline.

‡Ever reported between waves 1 and 5.

§Ever reported 1+ limitations with ADL.

¶Average between wave 0, wave 2 and wave 4.

Cognitive function domain scores are standardised to the z-score, mean 0 and SD 1. All predictors were entered simultaneously. Cells with dashes represent non-significant effects.

ADL, activities of daily living; BMI, body mass index; CVD, cardiovascular disease; SES, socioeconomic status.

Poor mobility was significantly related to a steeper decline in memory (−0.020, SE 0.010). Being physically inactive was related to faster decline in memory (−0.018, SE 0.009) and global cognitive function (−0.016, SE 0.007). Being a current smoker was only related to faster decline in processing speed and global cognitive function. Low education was related to a faster decline in memory (−0.026, SE 0.012), whereas low childhood socioeconomic status was related to a slower decline in global cognitive function (0.020, SE 0.007). After accounting for all risk factors, women showed significantly less decline in memory (0.011, SE 0.006, p<0.05), executive (0.012, SE 0.006, p<0.05) and global cognitive function (0.016, SE 0.004, p<0.001) than men (online supplementary table S1).

10.1136/jech-2017-210116.supp1 Supplementary data

### Sensitivity analyses

In supplementary materials (online supplementary tables S2–S5 and figures S1–S2), we report the results restricted to completers, that is, respondents who were present in all five measurement occasions (4157 individuals of which 2332 are women). In general, completers reported higher initial levels of cognitive function and slower rates of decline; fewer predictors were related to initial levels and rate of change in each cognitive domain; nevertheless, the overall conclusions remained unchanged.

Results of the sensitivity analysis for participants aged 60+ years at baseline are reported in online supplementary tables S6–S7. Smoking status was not related to decline in processing speed in this sample; among women, walking difficulties were not related to the decline in memory, whereas depression was found to be a significant predictor of executive function.

## Discussion

### Main results

In the present study, we examined age trajectories of cognitive function over an 8-year period and factors related to them in a nationally representative sample of older adults in England. There are two main findings. First, memory and executive function processing speed declined significantly over time, and the decline was steeper at older ages. Global cognitive function declined less rapidly in women compared with men. Second, we found several factors significantly related to initial levels of cognitive function, and fewer were predictive of cognitive decline. Age and a dementia diagnosis were related to a steeper decline in all cognitive function domains for both men and women. Depression predicted a steeper decline in executive and global cognitive function in men only. Physical inactivity was a predictor of decline in memory in women and global cognitive function in men and women. Processing speed and global cognitive function declined more steeply among women who were current smokers. For both men and women, limitations with ADL were related to a steeper decline in global cognitive function; additionally for women, poor mobility was related to a steeper decline in memory. Low education attainment was related to a steeper decline in memory for women; however, for global cognitive function, there was a positive effect of education on rate of change. Women in the lowest group of childhood socioeconomic status had significantly less decline in global cognitive function and memory, respectively, than those in the highest groups. After adjusting for all risk factors, women reported slower declines in memory, executive and global cognitive function than men.

### Comparisons with other studies

In accordance with previous studies, our results also showed that whereas most of the factors explored were related to initial levels of cognitive function, fewer of these predicted cognitive decline.^3 8 9^ For example, our results confirmed that increasing BMI was protective, although not strongly in magnitude, against lower initial levels of cognitive function, but no associations were found with cognitive decline.^3 9 32^ A possible explanation is that body fat contains leptin, which may be protective of poor cognitive function in old age.^33^ Another possible explanation is that in older people loss of lean body mass is common, therefore, higher lean body mass may reduce the risk of poor cognitive function.^34^

It has been suggested that moderate alcohol consumption is protective against poorer cognitive function and faster rate of cognitive decline, which might be mediated through cardiovascular risk factors.^14^ Similar to studies on the effect of moderate alcohol consumption on cognitive function,^35^ we found that daily alcohol consumption compared with less than daily was protective against poorer baseline cognitive function. However, in contrast with results of a British cohort study,^36^ we found that daily alcohol consumption was associated with faster decline in processing speed among men. Our result of faster rates of decline in processing speed and global cognitive function among women who were current smokers compared with never smokers is in accordance with a meta-analyses of the effect of smoking on cognitive function decline.^15^

We showed that wealth and education contributed to the initial levels of cognitive function as previously reported,^9 18^ but only to a faster rate of change of memory in women. Our results also showed that men in the lowest group of childhood socioeconomic status and women in the lowest education group had a slower decline in global cognitive function compared with those in the highest group. This could be partly due to the low initial level of cognitive function or to a confounding effect. To further investigate if this was the case, we conducted analyses in which predictors were entered one at the time, and we found that low childhood socioeconomic status and education were not significantly related to the slope of cognitive function (results available on request); however, when wealth, health and health behaviour variables were added into the model, the relationship became significant, suggesting that these factors were confounding the relationship between low childhood socioeconomic status, education and decline in cognitive function.

Poor physical functioning was only associated with the decline in global cognitive function among men and women and in memory among women. Several explanations can be hypothesised. First, we were not able to distinguish between those severely impaired from those less impaired. It is possible that only those who experience greater levels of physical impairment also experience more cognitive decline. Second, when we included gait speed, an objective measure of physical functioning, in the sample of those aged 60 years and over, in contrast with other studies,^37^ we found that this was not related to the rate of change of any domain of cognitive function. It is possible that, by using time-invariant variables, we were not able to detect the dynamic relationships between physical functioning and cognitive functioning. Results from a national sample of older adult participants of the Swedish Adoption/Twin Study of Ageing indicate that changes in pulmonary function,^29^ fine motor movement^38^ and grip strength^39^ were related to changes in cognitive function. In future work, we shall consider using similar time-varying measures of physical functioning.

Our results on gender differences in the rate of cognitive decline are in agreement with a recent study that showed that older women had greater resilience to age-related cognitive decline compared with men.^17^

### Strengths and limitations

The strengths of this study include the use of a large nationally representative sample of the English population aged 50 years and over with a long follow-up duration.^23^ This is the first study among the family of the USA’s HRS ageing studies to investigate concurrently factors related to three distinctive measures of cognitive function. Therefore, our findings make a unique contribution to the emerging longitudinal studies of ageing designed to be comparable with our study. A limitation of our study is that data on cognitive function were not collected before the age of 50 years, it is possible that the decline in cognitive function, and in particular of processing speed, occurred from younger ages. A second limitation is that each cognitive function domain was assessed by a single task; having multiple tests per domain is preferable. Third, we treated all covariates as time invariant; although there was no change over time in depression, smoking, alcohol consumption and physical activity, it is possible that changes in health and physical function over time were related to changes in cognitive function. This should be the subject of a future report. Another possible limitation is practice effects, common to all longitudinal studies of cognition. To further investigate the possibility of practice effects in our sample, we compared the average scores of people of the same age but who were taking the test for the first, second, third and so on occasion.^40^ We found minimal evidence of practice effects between the first and second occasion and no evidence of practice effects between the second and subsequent occasions. While attrition is a perennial source of bias in longitudinal studies of ageing, the results of our sensitivity analysis suggest that this did not unduly influence our results. Although we cannot entirely rule out that the missingness is purely at random, by ascertaining that the trajectory parameters were reasonably insensitive to variations in the values of missing data, we could conclude that the underlying reasons for missingness were not important.

## Conclusions

Our results have potentially important implications for the understanding of age-related cognitive decline and factors related to it. Assessing changes in cognitive function from mid-life onwards can help in identifying those who are at risk for progressing to mild cognitive impairment and those at risk of dementia. The health consequences of cognitive decline have been well documented, with studies reporting increased disability, decreased quality of life and loss of independence.^3^ This being the case, a better understanding of the determinants of cognitive decline is important.

**What is already known on this subject**

- Lower cognitive function and cognitive decline increase the risk of mortality, disability and poor quality of life. As such, understanding the natural history of age-related cognitive decline is important for early prevention.
- Although a few studies have explored predictors of cognitive decline in later life, results are inconclusive as to which factors could help in maintaining cognitive function.

**What this study adds**

- Using a nationally representative sample of older adults in England, we found that memory, executive function, processing speed and global cognitive function all declined significantly from early old age, and the decline was steeper at older ages.
- After adjustment, memory, executive and global cognitive function declined less rapidly in women compared with men.
- Age and dementia were related to a steeper decline in all cognitive function domains. Low education, poor physical functioning, depression and modifiable risk factors such as alcohol consumption, smoking and physical inactivity were all related to steeper decline in some cognitive function domains.
