# Supplementary material for: Cognitive function trajectories and their determinants in older people: 8 years of follow-up in the English Longitudinal Study of Ageing
Source: J Epidemiol Community Health. 2018 Apr 24;72(8):685–94. doi: 10.1136/jech-2017-210116 (PMC6204948; doi:10.1136/jech-2017-210116)
Supplement: Supplementary data [file jech-2017-210116supp001.pdf]

Cognitive function trajectories and their determinants in older people: 8 years of follow-up in the English Longitudinal Study of Ageing

Paola Zaninotto[1], G. David Batty[1,2], Michael Allerhand[2], Ian J. Deary[2]*

[1] *University College London, Research Department of Epidemiology and Public Health, London, United Kingdom*

[2] *Centre for Cognitive Ageing and Cognitive Epidemiology, Department of Psychology, University of Edinburgh, Edinburgh, UK*

*Correspondence to p.zaninotto@ucl.ac.uk

Table S1 Adjusted[a] estimates for the effect of gender on the intercepts and slopes of each cognitive function domain

	Estimate (s.e.)
**Memory Intercept**	0.194 (0.015)***
**Memory Slope**	0.011 (0.006)*
**Executive function Intercept**	-0.078 (0.017)***
**Executive function Slope**	0.012 (0.006)*
**Processing Speed Intercept**	0.324 (0.017)***
**Processing Speed Slope**	0.008 (0.006)
**Global CF Intercept**	0.145 (0.013)***
**Global CF Slope**	0.016 (0.004)***

a.Adjusted for age, education, wealth, and childhood socioeconomic status, cardiovascular disease, diabetes, limitations with activities of daily living, poor mobility, BMI, physical activity, alcohol, and smoking, depression, and dementia

.

Table S2. Summary characteristics of participants who completed all waves of the study (n=4157): the English Longitudinal Study of Ageing, 2002-2003 to 2010-2011

		Men (n= 1,825)		Women (n=2,332)
**Demographic**				
Age, y, Mean, (SD) range[a]	61.8 (8.1)	50-87	62.3 (8.5)	50-92
**Socioeconomic**				
Wealth[a]				
High	44.3	(42.1; 46.6)	38.6***	(39.6; 42.7)
Middle	35.5	(33.3; 37.4)	35.4	(33.4; 37.4)
Low	20.1	(18.4; 22.1)	26.1***	(24.3; 27.9)
Childhood SES[a]				
High	31.2	(29.1; 33.0)	33.9	(32.1; 35.9)
Middle	32.8	(30.7; 35.0)	30.0*	(27.8; 31.5)
Low	36.0	(33.8; 38.2)	36.5	(34.5; 38.4)
Education[a]				
High	21.3	(19.5; 23.3)	14.8***	(13.4; 16.2)
Middle	33.5	(31.4; 35.7)	38.3***	(36.4; 40.3)
Low	45.2	(42.9; 47.4)	46.9***	(44.9; 49.0)
**Health**				
CVD[b] % (95%CI)	24.7	(22.7; 26.7)	17.1***	(15.6; 18.7)
Diabetes[b] % (95%CI)	15.3	(13.7; 17.0)	10.9***	(9.7; 12.2)
**Physical functioning**				
ADL[b] % (95%CI)	32.1	(30.0; 34.2)	36.3**	(34.4; 38.3)
Poor mobility[b]	13.2	(11.7; 14.8)	16.7***	(15.2; 18.3)
**Health behaviours**				
BMI[c]	28.0	(27.8; 28.1)	28.1	(27.9; 28.3)
Current smoker[b]	16.6	(14.9; 18.3)	16.9	(15.4; 18.4)
Daily alcohol consumption[b]	47.2	(45.0; 49.5)	30.7***	(28.9; 32.6)
Physically inactive[b]	23.4	(21.5; 25.4)	36.5***	(34.5; 38.4)
**Depression**[b] % (95%CI)	23.0	(21.1; 25.0)	38.8***	(38.9; 40.1)
**Ever dementia**[b]	1.2	(0.7; 1.8)	1.1	(0.7; 1.6)
**Cognitive function measures**[b]	**Mean (SD)**	**Range**	**Mean (SD)**	**Range**
**Memory**				
Original score	10.2 (3.1)	1-20	10.6 (3.2)***	0-20
z-Transformed score	0.26 (0.86)	-2.3-3.0	0.35 (0.88)	-2.6-3.0
**Executive function**				
Original score	21.5 (6.2)	10-48	20.6 (5.9)***	0-44
z-Transformed score	0.35 (1.02)	-2.8-4.5	0.22 (0.92)	-3.0-3.9
**Processing speed**				
Original score	18.9 (5.2)	2-56	20.4 (5.4)***	0-63
z-Transformed score	0.05 (0.9)	-2.8-6.2	0.30 (0.9)	-3.1-7.5

a. Measured at baseline. b. Ever reported between waves 1 to 5. c. Average between wave 0, wave 2 and wave 4. *p<0.05 for the gender difference **p<0.01***p<0.001

Table S3. Results of the linear growth curve model for each cognitive function domain for the sample of completers, English Longitudinal Study of Ageing 2002-2003 to 2010-2011

	Memory (Standardized score)[a]		Executive (Standardized score)[a]		Processing speed (Standardized score)[a]		Global Cognitive function (Standardized score)[a]	
**Growth parameters**	**Estimate (se)**	**p-value**	**Estimate (se)**	**p-value**	**Estimate (se)**	**p-value**	**Estimate (se)**	**p-value**
Intercept	0.285 (0.017)	<0.001	0.351 (0.002)	<0.001	0.021 (0.019)	0.261	0.000 (0.000)	na
Intercept variance	0.296 (0.013)	<0.001	0.467 (0.016)	<0.001	0.418 (0.015)	<0.001	0.209(0.011)	<0.001
Slope	-0.023 (0.005)	<0.001	-0.017 (0.005)	<0.010	-0.050 (0.005)	<0.001	-0.032 (0.004)	<0.001
Slope variance	0.003 (0.001)	<0.001	0.012 (0.001)	<0.001	0.009 (0.001)	<0.001	0.003 (0.001)	<0.001
**Intercept on**								
Female	0.135 (0.023)	<0.001	-0.112 (0.026)	<0.001	0.295 (0.025)	<0.001	0.098 (0.020)	<0.001
Age	-0.031 (0.001)	<0.001	-0.026 (0.002)	<0.001	-0.019 (0.001)	<0.001	-0.030 (0.001)	<0.001
**Slope on**								
Female	0.013 (0.005)	<0.05	0.008 (0.007)	0.239	0.005 (0.006)	0.435	0.013 (0.005)	<0.010
Age	-0.005 (0.000)	<0.001	-0.004 (0.000)	<0.001	-0.004 (0.000)	<0.001	-0.005 (0.000)	<0.001
**Intercept and slope correlation**	0.011 (0.003)	<0.001	-0.003 (0.004)	0.349	-0.018 (0.003)	<0.001	0.007 (0.002)	0.001
**Model fit**								
CFI	0.988		0.995		0.995		0.975	
TLI	0.985		0.994		0.994		0.967	
RMSEA	0.041		0.026		0.026		0.036	
N	4,157		4,157		4,157		4,157	

a To the z-score, mean 0 and standard deviation 1. CFI = Comparative Fit Index. TLI = Tucker–Lewis index. RMSEA = root-mean-square error of approximation. Completers: those present at all measurement occasions with non-missing data on the CF tests at each given occasion.

Fig.S1 Ageing-vector graphs showing the predicted 8-year trajectories of memory, executive function, processing speed and global cognitive function for men completers (n=1,825), English Longitudinal Study of Ageing 2002-2003 to 2010-11.

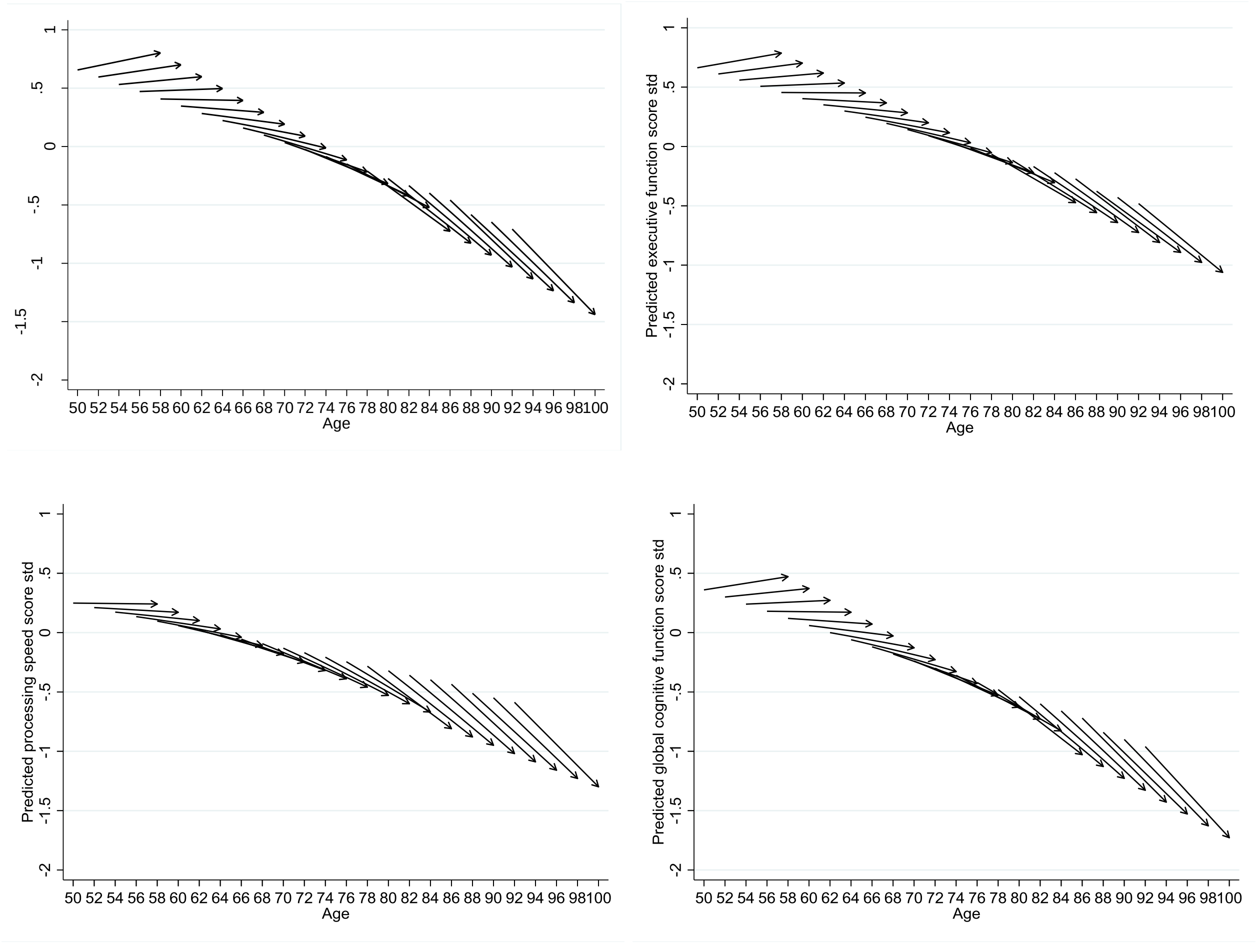

Fig.S2 Ageing-vector graphs showing the predicted 8-year trajectories of memory, executive function, processing speed and global cognitive function for women completers (n=2,332), English Longitudinal Study of Ageing 2002-2003 to 2010-11.

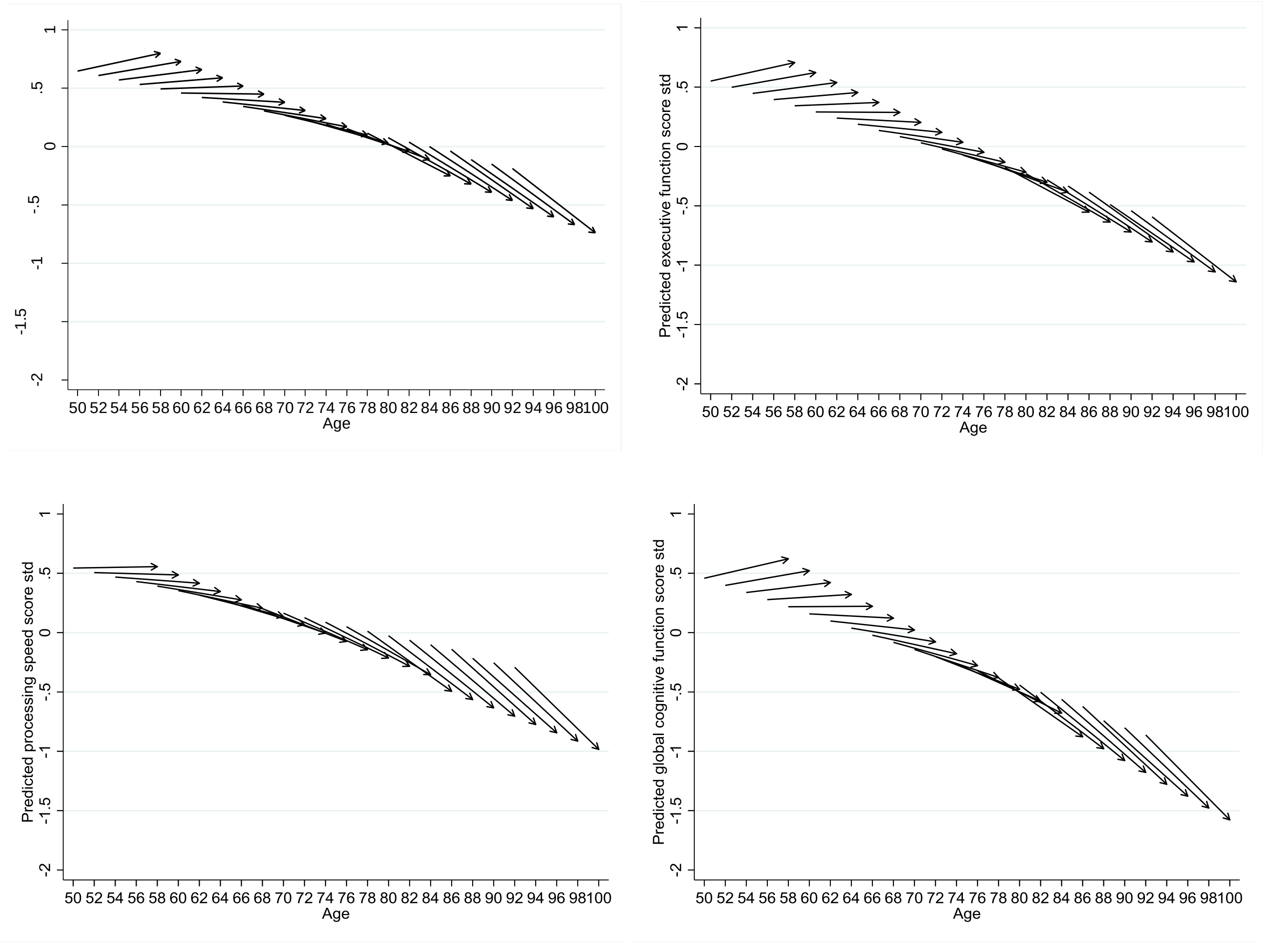

Table S4 Predictors of intercepts and slopes of change of each cognitive function domain for the completers men (n=1825), the English Longitudinal Study of Ageing, 2002-2003 to 2010-2011

Covariate	Memory Intercept	Memory Slope	Executive function Intercept	Executive function Slope	Processing Speed Intercept	Processing Speed Slope	Global CF Intercept	Global CF Slope
**Demographic**								
Age [a]	-0.026 (0.002)***	-0.004 (0.001)***	-0.020 (0.003)***	-0.004 (0.000)***	-0.017 (0.002)***	-0.002 (0.000)***	-0.025 (0.002)***	-0.004 (0.000)***
**Socioeconomic**								
Wealth (Middle)[a]	-	-	-	-	-0.137 (0.041)**	-	-	-
Wealth (Low)[a]	-0.130 (0.049)**	-	-	-	-0.158 (0.052)**	-	-0.123 (0.042)**	-
Childhood SES (Middle)[a]	-	-	-	-	-	-	-	-
Childhood SES (Low)[a]	-	-	-0.104 (0.052)**	0.042 (0.016)**	-	-	-	
Education (Middle)[a]	-0.106 (0.045)*	-	-0.176 (0.056)**	-	-0.158 (0.048)**	-	-0.147 (0.038)***	-
Education (Low)[a]	-0.389 (0.048)***	-	-0.419 (0.059)***	-	-0.274 (0.051)***	-	-0.413 (0.041)***	0.030 (0.009)**
**Health**								
CVD [b]	-	-	-	-	-	-	-	-
Diabetes [b]	-	-	-	-	-	-	-	-
**Physical functioning**								
ADL[b]	-	-	-0.110 (0.048)*	-	-	-	-0.073 (0.034)*	-
Poor mobility[b]	-	-		-	-	-	-	-
**Health Behaviors**								
BMI [c]	-	-	0.012 (0.005)*	-	-		-	-
Current smoker[b]	-	-	-	-	-	-	-	-
Daily alcohol drinking [b]	-	-	0.087 (0.053)*	-	-	-	0.062 (0.028)*	-
Physically inactive [b]	-0.147 (0.043)**	-	-	-	-	-	-0.136 (0.037)***	-
**Depression** [b]	-	-	-		-		-	-0.020 (0.009)*
**Dementia** [b]	-0.419 (0.148)**	-0.107 (0.043)*	-	-0.244 (0.050)***	-	-0.103 (0.042)*	-0.277 (0.126)*	-0.169 (0.032)***

***p<0.001 **p<0.01 *p<0.05. Cognitive function domain scores are standardized to the z-score, mean 0 and standard deviation 1. All predictors were entered simultaneously.
a Measured at baseline. b Ever reported between waves 1 to 5. c Average between wave 0, wave 2 and wave 4. Cells with dashes represent non-significant effects.

Table S5 Predictors of intercepts and slopes of change of each cognitive function domain for the completers women (n=2,332), the English Longitudinal Study of Ageing, 2002-2003 to 2010-2011

Covariate	Memory Intercept	Memory Slope	Executive function Intercept	Executive function Slope	Processing Speed Intercept	Processing Speed Slope	Global CF Intercept	Global CF Slope
**Demographic**								
Age [a]	-0.024 (0.002)***	-0.005 (0.001)***	-0.020 (0.002)***	-0.004 (0.001)***	-0.013 (0.002)***	-0.004 (0.001)***	-0.023 (0.002)***	-0.005 (0.001)***
**Socioeconomic**								
Wealth (Middle)[a]	-	-	-	-	-	-	-	-
Wealth (Low)[a]	-0.168 (0.041)***	-	-0.189 (0.046)***	-	-	-	-0.171 (0.09)***	-
Childhood SES (Middle)[a]	-	-	-	-	-	-	-	-
Childhood SES (Low)[a]	-0.137 (0.037)***	-	-0.153 (0.041)***	-	-0.127 (0.031)***	-	-0.154 (0.031)***	-
Education (Middle)[a]	-0.146 (0.0345)**	-	-0.277 (0.050)***	-	-	-	-0.194 (0.038)***	-
Education (Low)[a]	-0.397 (0.047)***	-	-0.527 (0.053)***	-	-0.164 (0.056)**	-	-0.440 (0.040)***	-
**Health**								
CVD [b]	-	-	-	-	-	-	-	-
Diabetes [b]	-	-	-	-	-	-	-	-
**Physical functioning**								
ADL[b]	-	-	-	-	-	-	-	-
Poor mobility[b]	-	-	-	-	-	-	-	-
**Health Behaviors**								
BMI [c]	-	-	0.007 (0.003)*	-	-	-	-	-
Current smoker[b]	-	-	-	-	-	-0.033 (0.012)**	-	-
Daily alcohol drinking [b]	0.066 (0.032)*	-	0.112 (0.036)**	-	-	-	0.083 (0.027)**	-
Physically inactive [b]	-0.085 (0.032)***	-	-0.149 (0.039)***	-	-0.092 (0.041)*	-	-0.118 (0.029)***	-
**Depression** [b]	-	-	-	-	-	-	-	-
**Dementia** [b]	-0.541 (0.142)***	-0.121 (0.042)**	-	-0.137 (0.045)**	-0.302 (0.072)***	-0.080 (0.0030)**	-0.421(0.118)***	-0.014 (0.031)*

***p<0.001 **p<0.01 *p<0.05. Cognitive function domain scores are standardized to the z-score, mean 0 and standard deviation 1. All predictors were entered simultaneously.
a Measured at baseline. b Ever reported between waves 1 to 5. c Average between wave 0, wave 2 and wave 4. Cells with dashes represent non-significant effects.

Table S6 Predictors of intercepts and slopes of change of each cognitive function domain for men aged 60 and over (n=3,025), the English Longitudinal Study of Ageing, 2002-2003 to 2010-2011

Covariate	Memory Intercept	Memory Slope	Executive function Intercept	Executive function Slope	Processing Speed Intercept	Processing Speed Slope	Global CF Intercept	Global CF Slope
**Demographic**								
Age [a]	-0.027 (0.002)***	-0.004 (0.000)***	-0.017 (0.002)***	-0.005 (0.002)***	-0.019 (0.002)***	-0.004 (0.001)***	-0.025 (0.002)***	-0.005 (0.001)***
**Socioeconomic**								
Wealth (Middle)[a]	-	-	-	-	-0.077 (0.036)*	-	-	-
Wealth (Low)[a]	-0.201 (0.039)***	-	-0.165 (0.042)***	-	-0.172 (0.038)***	-	-0.209 (0.033)***	-
Childhood SES (Middle)[a]	-	-	-	-	0.080 (0.038)*	-	-	-
Childhood SES (Low)[a]	-	-	-0.114 (0.040)**	-	-	-0.026 (0.013)*	-	
Education (Middle)[a]	-0.207 (0.047)***	-	-0.120 (0.051)*	-	-	-	-0.169 (0.040)***	-
Education (Low)[a]	-0.401 (0.046)***	0.041 (0.016) *	-0.270 (0.050)***	-	-0.219 (0.047)***	-	-0.357 (0.039)***	0.037 (0.013)**
**Health**								
CVD[b]	-	-	-	-	-	-	-	-
Diabetes[b]	-	-	-	-	-	-	-	-
**Physical functioning**								
ADL[b]	-0.234 (0.034)***	-	-0.219 (0.037)***	-	-0.087 (0.035)*	-	-0.222 (0.035)***	-
Poor mobility[b]	-		-		-		-	-
Gait speed[c]	0.607 (0.070)***	-	0.663 (0.076)***	-	0.545 (0.072)***	-	0.674 (0.060)***	-
**Health Behaviors**								
BMI[d]	0.009 (0.004)*		0.014 (0.004)**	-	-		0.010 (0.003)**	-
Current smoker[b]	-	-	-	-	-0.106 (0.041)*	-	-	-
Daily alcohol drinking[b]	0.078 (0.029)**	-	0.106 (0.031)***	-	0.086 (0.029)**	-	0.097 (0.024)***	-0.017 (0.008)*
Physically inactive[b]	-0.075 (0.033)*	-	-	-	-0.072 (0.034)*	-	-0.064 (0.028)***	-
**Depression**[b]	-	-	-	-	-	-	-	-0.021 (0.010)*
**Dementia**[b]	-0.405 (0.069)***	-0.173 (0.029)***	-0.205 (0.075)**	-0.225 (0.033)***	-0.289 (0.071)***	-0.089 (0.031)**	-0.355 (0.058)***	-0.196 (0.023)***

***p<0.001 **p<0.01 *p<0.05. Cognitive function domain scores are standardized to the z-score, mean 0 and standard deviation 1. All predictors were entered simultaneously. a Measured at baseline. b Ever reported between waves 1 to 5. c Average between wave 1-5. d Average between wave 0, wave 2 and wave 4. Cells with dashes represent non-significant effects.

Table S7 Predictors of intercepts and slopes of change of each cognitive function domain for women aged 60 and over (n=3,617), the English Longitudinal Study of Ageing, 2002-2003 to 2010-2011

Covariate	Memory Intercept	Memory Slope	Executive function Intercept	Executive function Slope	Processing Speed Intercept	Processing Speed Slope	Global CF Intercept	Global CF Slope
**Demographic**								
Age [a]	-0.036 (0.002)***	-0.004 (0.001)***	-0.022 (0.002)***	-0.004 (0.001)***	-0.025 (0.002)***	-0.003 (0.001)***	-0.032 (0.002)***	-0.005 (0.002)***
**Socioeconomic**								
Wealth (Middle)[a]	-	-	-	-	-	-	-0.061 (0.016)*	-
Wealth (Low)[a]	-0.160 (0.037)***	-	-0.150 (0.036)***	-	-0.098 (0.042)*	-	-0.161 (0.031)***	-
Childhood SES (Middle)[a]	-0.141 (0.035)**	-	-0.124 (0.034) **	-	-	-	-0.131 (0.029)**	-
Childhood SES (Low)[a]	-0.175 (0.035)***	-	-0.156 (0.034)***	-	-0.144 (0.039)***	-	-0.178 (0.029)***	0.024 (0.010)*
Education (Middle)[a]	-0.106 (0.047)**	-	-0.240 (0.046)***	-	-	-	-0.174 (0.039)***	-
Education (Low)[a]	-0.297 (0.047)***	-	-0.395 (0.046)***	-	-0.125 (0.054)*	-	-0.344 (0.040)***	-
**Health**								
CVD[b]	-	-	-	-	-	-	-	-
Diabetes[b]	-	-	-	-	-	-	-	-
**Physical functioning**								
ADL[b]	-0.154 (0.033)***	-	-0.148 (0.032)***	-	-0.102 (0.037)**	-	-0.159 (0.028)***	-0.019 (0.009)*
Mobility[b]	-	-	-	-	-	-	-	-
Gait speed[c]	0.587 (0.069)***	-	0.542 (0.067)***	-	0.595 (0.078)***	-	0.630 (0.058)***	-
**Health Behaviors**								
BMI[d]			0.009 (0.003)**	-	-		0.006 (0.002)*	
Current smoker[b]	-		-	-	-	-	-	-0.025 (0.011)*
Daily alcohol drinking[b]	0.070 (0.031)*	-	0.073 (0.030)*	-	-	-	0.063 (0.026)*	-
Physically inactive[b]	-	-0.034 (0.009)**	-	-0.027 (0.012)*	-	-	-	-0.029 (0.009)**
**Depression**[b]	-	-	-	-0.021 (0.010)*	-	-	-	-0.017 (0.008)*
**Dementia**[b]	-0.476 (0.063)***	-0.183 (0.028)***	-0.239 (0.062)***	-0.209 (0.028)***	-0.302 (0.072)***	-0.080 (0.031)**	-0.393 (0.053)***	-0.201 (0.022)***

***p<0.001 **p<0.01 *p<0.05. Cognitive function domain scores are standardized to the z-score, mean 0 and standard deviation 1. All predictors were entered simultaneously. a Measured at baseline. b Ever reported between waves 1 to 5. c Average between wave 1-5. d Average between wave 0, wave 2 and wave 4. Cells with dashes represent non-significant effects.
